# Cumulative Effect of Loneliness and Social Isolation on Health Outcomes among Older Adults

**DOI:** 10.1093/geroni/igab046.3310

**Published:** 2021-12-17

**Authors:** Timothy Barnes, Stephanie MacLeod, Rifky Tkatch, Manik Ahuja, Laurie Albright, James Schaeffer, Charlotte Yeh

**Affiliations:** 1 UnitedHealth Group, Minnetonka, Minnesota, United States; 2 UnitedHealth Group, United States; 3 UnitedHealth Group, Oak Park, Michigan, United States; 4 UnitedHealth Group, Minneapolis, Minnesota, United States; 5 AARP Services, Inc., Washington, District of Columbia, United States

## Abstract

Loneliness and social isolation are described similarly yet are distinct constructs. Numerous studies examine each construct separately; however, less research has been dedicated to exploring their impacts together. Using survey and claims data among adults age 65+ (N=6,994), the cumulative effects of loneliness and social isolation on late-life health outcomes were examined using Chi-square and multivariate regression models. Loneliness and social isolation were measured using the UCLA-3 Loneliness Scale and the Social Network Index. Participants were grouped into four categories of loneliness and social isolation based on overlap, including: lonely only (L), socially isolated only (SI), both lonely and socially isolated (LSI), or neither (N). Outcomes included quality of life and healthcare utilization and costs. Among participants, 9.8% were considered L, 20.6% SI, 9.1% LSI, and 60.5% N. Respondents were primarily female (55.0%) and 70-74 years of age (27.1%). Those considered LSI were more likely to be older, female, less healthy, depressed, with lower quality of life and greater healthcare utilization patterns. Participants who were L or LSI had higher rates of emergency room visits compared to the N group; LSI had the highest medical costs. Results demonstrate the cumulative effects of loneliness and social isolation among older adults. Findings not only fill a gap in research exploring the impacts of these constructs later in life, but also confirm the need for approaches targeting older adults who are both lonely and socially isolated. As the COVID-19 pandemic continues, this priority will continue to be urgent for older adults.

